# Probing the Run-On Oligomer of Activated SgrAI Bound to DNA

**DOI:** 10.1371/journal.pone.0124783

**Published:** 2015-04-16

**Authors:** Santosh Shah, Jonathan Sanchez, Andrew Stewart, Michael M. Piperakis, Richard Cosstick, Claire Nichols, Chad K. Park, Xin Ma, Vicki Wysocki, Jurate Bitinaite, Nancy C. Horton

**Affiliations:** 1 Department of Chemistry and Biochemistry, University of Arizona, Tucson, AZ, 85721, United States of America; 2 Genetics Graduate Program, University of Arizona, Tucson, AZ 85721, United States of America; 3 Department of Chemistry, University of Liverpool, Liverpool, United Kingdom, L69 7ZD, United States of America; 4 Department of Chemistry and Biochemistry, Ohio State University, Columbus, OH 43210, United States of America; 5 New England Biolabs, Inc., 240 County Road, Ipswich, MA 01938, United States of America; University of Quebect at Trois-Rivieres, CANADA

## Abstract

SgrAI is a type II restriction endonuclease with an unusual mechanism of activation involving run-on oligomerization. The run-on oligomer is formed from complexes of SgrAI bound to DNA containing its 8 bp primary recognition sequence (uncleaved or cleaved), and also binds (and thereby activates for DNA cleavage) complexes of SgrAI bound to secondary site DNA sequences which contain a single base substitution in either the 1^st^/8^th^ or the 2^nd^/7^th^ position of the primary recognition sequence. This modulation of enzyme activity via run-on oligomerization is a newly appreciated phenomenon that has been shown for a small but increasing number of enzymes. One outstanding question regarding the mechanistic model for SgrAI is whether or not the activating primary site DNA must be cleaved by SgrAI prior to inducing activation. Herein we show that an uncleavable primary site DNA containing a 3’-*S*-phosphorothiolate is in fact able to induce activation. In addition, we now show that cleavage of secondary site DNA can be activated to nearly the same degree as primary, provided a sufficient number of flanking base pairs are present. We also show differences in activation and cleavage of the two types of secondary site, and that effects of selected single site substitutions in SgrAI, as well as measured collisional cross-sections from previous work, are consistent with the cryo-electron microscopy model for the run-on activated oligomer of SgrAI bound to DNA.

## Introduction

SgrAI, a type II restriction endonuclease, appears to be a member of a growing list of enzymes that form filaments or run-on oligomers with modulated (activated or inhibited) enzymatic activities [1]. This list currently also includes members such as IRE1, an RNA splicing nuclease/protein kinase involved in the unfolded protein response [2], acetyl-CoA carboxylase (ACC)[3], and CTP synthase (CTPS) [4]. Unlike amyloid, these run-on oligomers are transient, reversible, and activate (or inhibit) enzyme activity. In the case of SgrAI, the rate of DNA cleavage has been shown to be enhanced over 200 fold in the run-on oligomeric form, and its DNA specificity expanded to include other sequences (secondary site sequences) in addition to its primary target site sequence [5,6]. Of the other members of this novel group, only IRE1 also shows expansion of specificity in addition to activation in the run-on oligomeric form, in its ability to cleave other mRNA as well as its primary target (transcription factor XBP1 mRNA), leading to different biological outcomes (apoptosis vs. stress response) [7]. The proposed biological roles for run-on oligomerization by the different enzymes include rapid activation (or inactivation), storage of inactive enzyme, increase of substrate affinity through larger binding surfaces, and uniquely to SgrAI, sequestration of activated SgrAI on invading phage DNA (which protects the host bacterial genome from secondary site cleavage by activated SgrAI) [8–10].

The x-ray crystal structure of SgrAI has been solved in the low activity, dimeric form bound either to primary or secondary site DNA [11,12]. The structure of the run-on oligomeric form, argued to be the activated form of SgrAI [5], has been described at 8.6 Å using single particle cryo-EM (cryo-EM) reconstruction [10], and shows the association of DNA bound SgrAI dimers in a left-handed helix with contacts between neighboring SgrAI/DNA complexes that include both protein-protein and protein-DNA contacts. The protein-DNA contacts between neighboring complexes occur to the base pairs which flank the recognition site, an observation that explains the dependence of activation of SgrAI on the length or number of these flanking base pairs [5,10,13].

Activation of DNA cleavage by SgrAI has been studied using an assay with a low concentration (i.e., 1 nM) of radiolabeled DNA containing the recognition site, a high concentration of SgrAI (i.e., 1 μM), and varied concentration of added unlabeled activator DNA (0–2 μM). The increasing concentration of activator DNA in the presence of excess SgrAI results in an increasing concentration of SgrAI bound to activator DNA. This in turn results in their increasing association into the run-on oligomer, which binds and thereby activates SgrAI bound to the reporter DNA, and consequently increases the observed rate constant for cleavage of DNA by SgrAI [5]. The activator DNA used in the assay is typically a pre-cleaved version of an SgrAI primary site sequence embedded in a DNA with 16 flanking bp, known as PC (for pre-cleaved) DNA. Earlier studies observed that the pre-cleaved primary site DNA (which may be synthetic DNA identical to those generated from cleavage by SgrAI) activates DNA cleavage by SgrAI [6]. Additional studies suggested that the DNA need not be pre-cleaved in order to activate SgrAI and the pre-cleavage is not required for formation of the run-on oligomer [5]. However conclusive proof that the uncleaved form could be activating was lacking since a small fraction of DNA cleavage could in principle lead to large scale activation as a result of oligomer formation (i.e., rapid allosteric communication to a large number of SgrAI/DNA complexes by a small number of complexes containing cleaved DNA in a single SgrAI/DNA oligomer).

Herein we investigate the requirement for DNA cleavage of primary site DNA for activation of SgrAI by using a noncleavable 3’-*S*-phosphorothiolate substituted primary site DNA. We also re-investigate the cleavage of secondary site DNA, using DNA constructs containing longer flanking base pairs, as well as investigate the differences between the two types of secondary site DNA (where substitutions occur at either the 2^nd^/7^th^ or the 1^st^/8^th^ position of the recognition sequence). In addition, we compare the DNA cleavage results with secondary site DNA to those with a noncognate DNA that contains two substitutions, rather than only one, in the recognition sequence. Finally, we investigate the effects of several single site substitutions designed to disrupt predicted protein-DNA interfaces found in the cryo-EM model of the activated, run-on oligomer of SgrAI bound to DNA [10], as well as compare the results of previous measures of the run-on oligomer utilizing two distinct structural methods [9,10].

## Materials and Methods

### Mutagenesis and Protein Purification

Details of mutagenesis and protein purification may be found in S1 Methods and S2 Methods

### DNA Preparation

**Fig 1:**
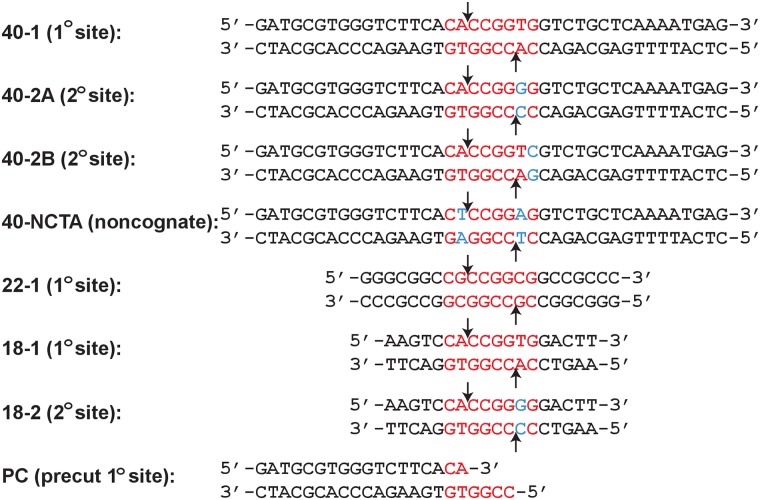
DNA sequences used in assays: SgrAI recognition sequences are shown in red, variations in secondary site sequences are shown in cyan, and arrows indicate the cleavage sites. The 3’S-phosphorothiolate substitution in 22-1-3’S occurs at the cleavage site on both strands of 22–1.

The oligonucleotides were made synthetically and purified using C18 reverse phase HPLC or denaturing PAGE[14]. The concentration was measured spectrophotometrically, with an extinction coefficient calculated from standard values for the nucleotides[15]. The self-complementary DNA strands, or equimolar quantities of complementary DNA, were annealed by heating to 90°C for 10 minutes at a concentration of 0.1–1 mM, followed by slow-cooling to 4°C over 4–5 hours in a thermocycler or heat block. Sequences of the DNA used are shown in Fig 1.

Because freeze-thawing alters the concentration of double stranded DNA used in the assays, DNA used for single turnover assays was treated very carefully to minimize this problem. Such DNA samples were aliquoted into small amounts, flash frozen in liquid nitrogen, stored at -20°C, and used only once after removing from the freezer. The 5’ ^32^P end labeling of DNA was performed with T4 polynucleotide kinase (New England Biolabs, Ipswich, MA, USA) and [γ-^32^P]-ATP (Perkin-Elmer, Inc., Waltham, MA, USA), followed by removal of excess ATP using P-30 spin columns (Bio-Rad Laboratories, Inc., Hercules, CA, USA).

The oligonucleotide containing 2’-deoxy-3'-thioguanosine (sulfur replacing 3'-oxygen at the cleavage site) was prepared from *N*2-isobutyryl-5′-dimethoxytrityl-2′-deoxy-3′-thioguanosine-3′-*S*-[(2-cyanoethyl)-(*N*,*N*-diisopropyl)]-phosphoramidite[16] and assembled on a MerMade 4 synthesizer (AME Bioscience, Toroed, Norway). The thioamidite (0.2 M concentration) was incorporated into oligodeoxynucleotides using 5-ethylmercapto-1H-tetrazole (1 M) as the activator as previously described[16–18]. A minor alteration to the reported coupling procedure is that two sequential 5 min coupling reactions were performed with an oxidation step between the coupling reactions. Oligonucleotides were purified by reverse phase HPLC (Phenomenex Gemini, Torrance, CA, USA, 5μm C-18 column) with the dimethoxytrityl (DMT) group “on” using a gradient of 0–40% acetonitrile in aqueous triethylammonium bicarbonate (50 mM) and the DMT group finally removed as described. MALDI-TOF spectra of the phosphorothiolate containing oligonucleotide were recorded using a Voyager DE-STR spectrometer supplied from Applied Biosystems (Life Technologies, Grand Island, NY, USA) with linear and reflection analyzers. The sample (~ 30 μM) was mixed in a 1:10 ratio with the matrix (matrix = 3-hydroxypicolinic acid; 1:1MeCN:H_2_O). Spectra were run in positive polarity, linear mode, with positive ion detection. The theoretical mass of the 3′-S oligonucleotide = 6756 and the measured mass = 6755 [M+H]^+^. The modified DNA was subsequently gel purified for use in the DNA cleavage assays.

### Single Turnover DNA Cleavage Assay

Single turnover kinetic measurements of DNA cleavage were performed using 5′ ^32^P-labeled oligonucleotides substrates (1 nM), under conditions of excess enzyme (1 μM SgrAI dimer), with and without the addition of unlabeled DNA. For reactions using 40-2A-Top and 40-2A-Bottom, the top or bottom strands respectively were individually labeled before being annealed with the unlabeled complementary strand. For all other reactions the complementary strands were annealed before being labeled. All DNA cleavage reactions were performed at 37°C in 20 mM Tris-HOAc (pH 8.0@RT), 50 mM KOAc, 10 mM Mg(OAc)_2_, and 1 mM DTT. 5 μl aliquots were withdrawn at specific time intervals after mixing the enzyme and labeled DNA (100 μl total reaction volume), quenched by addition to 5 μl of quench solution (80% formamide, 50 mM EDTA, 1 mg/ml XCFF dye, and 1 mg/ml BPB dye), and electrophoresed on denaturing polyacrylamide gels (20% acrylamide:bisacrylamide (19:1 ratio), 4 M urea, 89 mM Tris base, 89 mM boric acid, 2 mM EDTA). Autoradiography of gels was performed without drying using a phosphor image plate exposed at 4°C for 12–17 hours. Densitometry of phosphor image plates was performed with a phosphorimager (GE Healthcare Life Sciences, Pittsburgh, PA, USA or Bio-Rad, Inc., Hercules, CA, USA), and integration using ImageQuant (GE Healthcare Life Sciences, Pittsburgh, PA, USA), ImageJ[19], or Image Lab software (Bio-Rad, Inc., Hercules, CA, USA). The percent of product formed as a function of time was determined by integrating the density of both cleaved and uncleaved DNA bands, and normalizing to the total amount cleaved. The percentage of cleaved DNA was then fitted to a single exponential function to determine the single turnover rate constant of DNA cleavage using Kaleidagraph (Synergy Software, Reading, PA, USA):

$$\text{Percentage of product}={\text{ C}}_{1}+{\text{ C}}_{2}*\left(1-{\text{e}}^{-\text{kt}}\right)$$

where C_1_ is a constant fitting the baseline, C_2_ is the total percent of DNA predicted to be cleaved by SgrAI, k is the cleavage rate constant, and t is the length of incubation in minutes. The data from some reactions fit poorly to only one single exponential function, suggesting multiple phases. These were found to fit well to the sum of two single exponential functions:

$$\text{Percentage of product}={\text{ C}}_{1}+{\text{C}}_{2}*\left(1-{\text{e}}^{-{\text{k}}_{1}\text{t}}\right)+{\text{C}}_{3}*\left(1-{\text{e}}^{-{\text{k}}_{2}\text{t}}\right)$$

where C_1_ is a constant fitting the baseline, C_2_ is the total percent of DNA predicted to be cleaved by SgrAI with rate constant k_1_, C_3_ is the total percent of DNA predicted to be cleaved by SgrAI with rate constant k_2_, and t is the length of incubation in minutes. Measurements were performed at least three independent times, and presented as the average±standard deviation.

### DNA binding assays

The gel shift assay[20] was used to measure the binding affinity of S56E and A57E variants of SgrAI to PC DNA. The PC DNA was 5’ end labeled with ^32^P and held constant at a concentration of 0.1 nM (S56E) or 1 nM (A57E) in 20 μl binding buffer: 20 mM Tris-HOAc (pH 8.0@RT), 50 mM KOAc, 10 mM Ca(OAc)_2_, 1 mM DTT, 10% glycerol. Separate incubations were performed with the DNA and increasing concentrations of SgrAI enzyme (0.1 nM—1 μM enzyme dimer). These concentrations were chosen carefully to give a well-defined binding curve. Native polyacrylamide gel electrophoresis (8% 29:1 acrylamide:bisacrylamide, 89 mM Tris base, 89 mM boric acid, and 10 mM Ca^2+^) was used to separate the bound and unbound DNA. Care was taken to prevent heating of the gel, by running at 4°C at low voltage (190V). The electrophoresis buffer (89 mM Tris base, 89 mM boric acid, and 10 mM Ca^2+^) was recirculated during electrophoresis. Gels were loaded while undergoing electrophoresis at 300 V, and the voltage returned to 190 V five minutes after the loading of the last sample. Gels were then electrophoresed an additional 2–3 hours at 4°C. Autoradiography of gels was performed as described above in the *Single Turnover DNA Cleavage Assay* section. As the stoichiometries of binding of the SgrAI dimer to DNA containing the SgrAI recognition site [5], and to PC DNA (see below)[9] have been previously determined, the equilibrium dissociation constant, K_D_, was determined by fitting of the equation derived for 1:1 binding using the software Kaleidagraph (Synergy Software, Reading, PA, USA)[21]:

A=Amin+(Amax−Amin)[(PT+OT+KD)−[(PT+OT+KD)2−(4PTOT)]12]/(2OT)

where A is the integrated density of the shifted DNA band at a given protein concentration, A_max_ is the fitted density corresponding to the integrated density of the shifted DNA band when all DNA is bound, A_min_ is the fitted density corresponding to the integrated density of the shifted DNA band in incubations without SgrAI, P_T_ is the total concentration of SgrAI, O_T_ is the total concentration of the DNA, and K_D_ is the dissociation constant to be determined. All measurements were performed in triplicate. Because previous data indicate that at 4°C the SgrAI dimer binds to two annealed copies of PC DNA, the O_T_ concentration is adjusted to half the concentration of PC DNA [5].

The equilibrium dissociation constant (K_D_) for the R131A and R134A variants of SgrAI and PC DNA, as well as for wild type SgrAI and 40-2A, 40-2B, and 40-NCTA was measured using fluorescence anisotropy [5,21–23]. Fluorescein 5’ end labeled (labeled on top strand only) PC, 40-2A, 40-2B, or Hex labeled 40-NCTA DNA at 1 nM in 2 ml of binding buffer (20 mM Tris-HOAc (pH 8.0@RT), 50 mM KOAc, 10 mM Ca(OAc)_2_, 1 mM DTT, with 10% glycerol (R131A, R134A SgrAI and PC DNA) or without glycerol (wild type SgrAI/40-NCTA/40-2A/40-2B)) at 4°C was titrated with increasing amounts of SgrAI enzyme (1–200 nM SgrAI dimer), and the anisotropy of the emitted fluorescence monitored. In addition, the K_D_ was also measured for SgrAI and 40-2B in buffer containing Mg^2+^ in place of Ca^2+^ (20 mM Tris-HOAc (pH 8.0@RT), 50 mM KOAc, 10 mM Mg(OAc)_2_, 1 mM DTT). Excitation occurred at 494 nm (fluorescein) or 533 nm (hexachlorofluorescein) in a PC1 (ISS Inc., Champaign, IL, USA) fluorimeter with temperature control. The emitted intensities were measured using a 50.8 mm diameter 570 nm cutoff filter with a 580–2750 nm transmittance range (ThermoOriel Inc., Stratford, CT, USA, catalog no. 59510) and 1 mm slit widths. The anisotropy of the emitted light as a function of added enzyme was fitted to the binding model as described above for the gel shift assay, with the exception that A is the anisotropy at a given protein concentration, A_max_ is the predicted anisotropy of fully bound DNA, A_min_ is the anisotropy with no protein binding. Measurements were repeated in triplicate.

### Collisional Cross-section (CCS) calculation

Theoretical CCSs were calculated using MOBCAL[22] and the scaled PA method developed by the Robinson group[23] using the model for the run-on oligomer derived from cryo-EM[10]. Computations of CCSs with MOBCAL were performed on a 2.83 GHz quad-core Xeon SGI Altix ICE 8200 (Fremont, CA, USA) server at the University of Arizona.

## Results

### Uncleaved primary site DNA activates SgrAI

Previous studies show that activation of SgrAI was possible with either intact or with pre-cleaved primary site DNA added to the DNA cleavage assays, however it was not known if the uncleaved DNA itself was the activator, or if it must be cleaved first to become the activator [5,6]. To test whether cleavage of the primary site DNA is a prerequisite for its ability to activate SgrAI, a noncleavable oligonucleotide with a 3’-*S*-phosphorothiolate substitution (22-1-3’S) at the site of cleavage by SgrAI was prepared (Materials and Methods, Fig 1). Addition of the nonsubstituted version of this oligonucleotide (22–1, Fig 1) to cleavage assays containing 1 nM ^32^P labeled 18 bp primary site containing DNA (^32^P-18-1, Fig 1) and 1 μM SgrAI dimer, induces activation of SgrAI resulting in an observed rate constant of 1.1±0.4 min^-1^ (Table 1), an approximate 10 fold increase over that in the absence of 22–1 DNA [10]. The 3’S modification was confirmed to block cleavage of 22-1-3’S DNA by SgrAI, even in the presence of 1 μM 22-1-3’S or 1 μM PC DNA (S1 Results, S1 Fig), as expected. However, in standard assays this modified DNA was found to be capable of inducing activation of ^32^P-18-1 cleavage by SgrAI (at 1 μM 22-1-3’S)(S2 Fig), resulting in a rate constant of 1.47±0.12 min^-1^, a 16 fold increase over unstimulated cleavage (0.094±0.015 min^-1^)[10](Table 1). Therefore this data shows that cleavage of a primary site DNA is not required for its ability to activate SgrAI, and intact primary sites do in fact activate SgrAI.

**Table 1:** Single-Turnover DNA Cleavage Rate Constants (k_obs_) using 1 μM SgrAI dimer.

^32^P-labeled Reporter DNA (1 nM)	Added Unlabeled Activator DNA	k_obs_ ^*a*^ (min^-1^)^*a*^	Percent Cleaved^*a*^ ^,^ ^*b*^	Relative Acceleration^*c*^
18–1	0 nM PC^*d*^	0.094±0.015	-	-
	10 nM PC^*d*^	0.18±0.06	-	2
	100 nM PC^*d*^	0.30±0.03	-	3
	250 nM PC	12.6±0.8	74±4	130
	500 nM PC	27±7	90±10	300
	1000 nM PC^*d*^	22±7	-	230
	1000 nM 22–1^e^	1.1±0.4		12
	1000 nM 22-1-3’S	1.47±0.12	-	16
40–1	0 nM PC	0.236±0.006	68±1	-
	10 nM PC	0.37±0.01	71±1	1.5
	100 nM PC	4.4±0.4	69±2	20
	250 nM PC	11.0±0.3	71.6±0.4	50
	500 nM PC	18.0±0.3	75.8±0.2	80
	1 μM PC	16.3±1.7	76.3±0.4	70
	2 μM PC	23±2	73.0±0.8	98
40–1	1 nM 40–1	0.28±0.02	63±2	-
	1 μM 40–1	12±1	29±3	40
40-2A	0 nM PC	0.013±0.004	17.7±0.5	-
	10 nM PC	0.018±0.004	70±7	1
	100 nM PC	0.039±0.005	63±2	3
	250 nM PC	0.82±0.09	28±2	60
	500 nM PC	6.1±0.6	53±1	500
	1 μM PC	9±3	53±7	700
	2 μM PC	10±3	38±3	800
40-2A	1 nM 40-2A	0.004±0.003	34±12	-
	1 μM 40-2A	0.25±0.06	78±5	20
40-2B	100 nM PC	0.0221±0.0009	54±3	2^*f*^
	250 nM PC	1.23±0.07	20±4	100^*f*^
	500 nM PC	2.2±0.4	29±2	170^*f*^
	1 μM PC	8.1±1.4	33±2	600^*f*^
	2 μM PC	11.8±1.4	45±2	900^*f*^
40-2B-Top	0 nM PC	0.0129±0.0004	69.7±0.2	-
	1 μM PC	9.3±1.6	55.2±0.3	700
40-2B- Bottom	0 nM PC	0.0023±0.0009	46±17	-
	1 μM PC	17±4	50±1	7000
40-NC-TA	0 nM PC	0.024±0.004	11±1	-
	1 μM PC	0.052±0.006	5.2±0.6	2

^*a*^Values presented at the average of three independent trials ± the standard deviation.

^*b*^Percent Cleaved is the percentage of DNA cleaved by SgrAI in the assay by the given rate constant (k_obs_). In cases where less than 100% is cleaved by the given rate constant, the remainder is cleaved by a nonaccelerated rate constant.

^*c*^Relative Acceleration is the ratio of k_obs_ to that in the absence of added unlabeled DNA.

^*d*^From Park, *et al*., 2010[8].

^*e*^ From Park, *et al*., 2010[10].

^*f*^Relative to cleavage of 40-2B-Top with no PC DNA.

### Cleavage of 18–1 DNA in the presence of 250 and 500 nM PC DNA

Previously, we reported the observed single turnover rate constants for cleavage of 1 nM ^32^P-18-1 DNA by 1 μM SgrAI dimer in the presence of 10, 100, and 1000 nM activator PC DNA[5]. These data showed very limited activation of SgrAI-mediated DNA cleavage in the presence of 10 and 100 nM PC (0.18±0.06 min^-1^ and 0.30±0.03 min^-1^, respectively, representing only 2 and 3 fold activation relative to no added PC DNA), but over 200 fold activation occurred in the presence of 1000 nM PC (22±7 min^-1^, 230 fold activation). However, no data were available at concentrations of PC DNA between 100 and 1000 nM PC DNA. Therefore, single turnover DNA cleavage reactions were performed with 250 and 500 nM PC DNA (Table 1). These data show that activation of SgrAI is considerable even at 250 nM PC (12.6±0.8 min^-1^, 130 fold activation), and activation at 500 nM PC (27±7 min^-1^, 300 fold activation) was comparable to that at 1000 nM PC.

### Cleavage of primary and secondary site embedded in 40 bp DNA

Single turnover DNA cleavage assays of primary, secondary, and noncognate site embedded in a 40 bp-long DNA were performed with 1 nM ^32^P labeled DNA, 1 μM SgrAI dimer, and varied concentrations of added unlabeled stimulatory PC DNA (DNA sequences for constructs are shown in Fig 1, rate constants shown in Table 1). In the case of the primary site DNA, 40–1, and without added PC DNA, the cleavage data fit well to a single exponential function with a rate constant of 0.236±0.006 min^-1^. With the addition of PC DNA, fitting of the cleavage data required two single exponential functions, suggestive of two independent processes (as described previously[5]). When two rate constants were fitted to the data, the slower was found to be similar to a non-accelerated reaction, and therefore only the faster of the two is given in Table 1 (along with the percentage of DNA cleaved with the accelerated rate constant). It was found that the majority of the 40–1 DNA was cleaved with the faster of the two rate constants, which ranged from 0.37±0.01 min^-1^ at 10 nM PC DNA to 23±2 min^-1^ with 2 μM PC DNA, representing up to a ~100 fold increase over cleavage without the added PC DNA. To test the stimulatory capacity of the 40–1 DNA itself, unlabeled 40–1 was added to the reactions at 1 μM. In this case, a large proportion of the DNA was cleaved with a single turnover rate constant of 12±1 min^-1^, a ~40 fold increase over the unstimulated cleavage rate constant.

**Fig 2:**
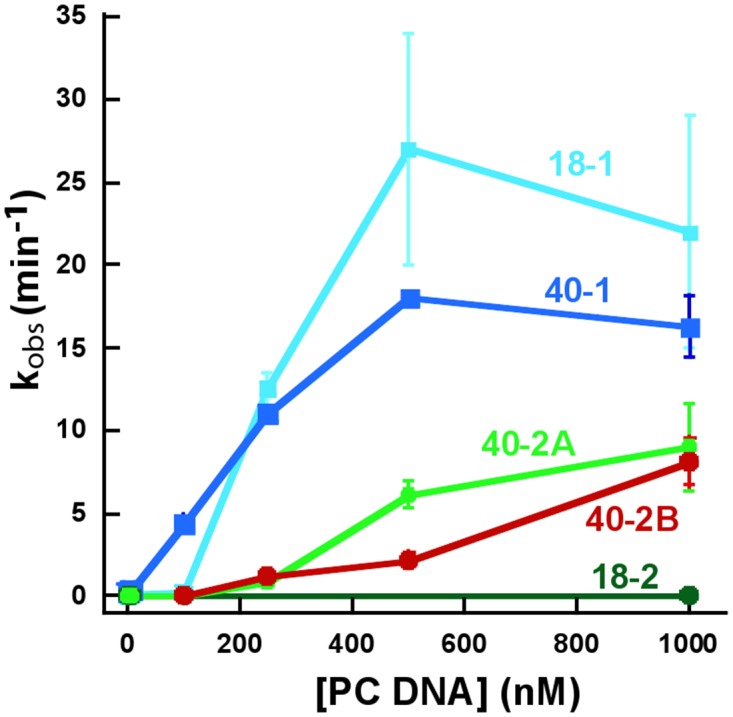
Plot of kobs (rate constants for cleavage) of 1 nM 32P labeled 18–1 (cyan filled squares), 40–1 (dark blue filled squares), 40-2A (green filled circles), 40-2B (red filled circles) and 18–2 (dark green filled circles) with 1 μM SgrAI and varied concentration of activator PC DNA. Data for 18–2 from Park, *et al*., 2010[5]. Lines are drawn to aide in visualization of the data.

Previously reported data of secondary site cleavage by SgrAI in the presence of activating PC DNA utilized a secondary site sequence embedded in an 18 bp DNA, and only very low levels of activation (2–3 fold) were observed[5]. Here, we measured the cleavage of two different types of secondary site in the context of a 40 bp DNA (40-2A and 40-2B, Fig 1) by SgrAI (Table 1). 40-2A has the sequence CA|CCGGGG/CC|CCGGTG (| denotes cut site), where the substitution of the primary site (i.e. CR|CCGGYG, R = A or G, Y = C or T) occurs in one of the two degenerate base pair positions at the 2^nd^/7^th^ position (underlined). In the absence of added PC DNA, the cleavage rate constant of 40-2A by SgrAI is very slow (0.013±0.004 min^-1^), and the percentage of DNA cleaved is only 17.7±0.5%. However, the addition of 500 nM PC DNA to the reaction results in an increase of the cleavage rate constant to 6.1±0.6 min^-1^, a 500 fold increase, and the total amount of DNA cleaved also increases to nearly 80% (Table 1). Further, in the presence of 2 μM PC DNA, SgrAI cleaves 40-2A DNA with a rate constant of 10±3 min^-1^, representing a ~1000 fold increase from the unstimulated cleavage rate with this DNA. Thus this data shows that when embedded in a longer DNA (i.e. 40 bp), the cleavage of the secondary site can be accelerated to levels similar to that of primary site DNA (Fig 2).

Single turnover DNA cleavage assays were also carried out using the second type of secondary site DNA, CACCGGTC/GACCGGTG, which contains a single substitution on each strand in the 1^st^/8^th^ position of the recognition sequence. This construct, 40-2B, contains the same flanking DNA as 40–1 and 40-2A (Fig 1). The accelerated DNA cleavage rate constants (k_obs_) of 1 nM 40-2B by 1 μM SgrAI in the presence of 1 and 2 μM PC were determined to be 8.1±1.4 min^-1^ and 11.8±1.4 min^-1^ respectively (Table 1), representing accelerations of 600 and 900 fold. These values are also very similar to those found for 40-2A, as described above (Table 1). Interestingly, in the absence of unlabeled PC DNA, very little cleavage of the bottom strand of 40-2B is observed. The measured rate constants of the top and bottom strands of 40-2B (Fig 1) in the absence of PC DNA (Table 1) show that the top strand is cleaved ~5-fold faster than the bottom strand. However, in the presence of 1 μM PC, both strands are cleaved with similar rate constants, which are similar to those of 40-2A (see 500 nM PC DNA, Table 1). Comparing the cleavage rate constants of the two types of secondary sites at intermediate concentrations of PC DNA shows that those of 40-2A are greater than those of 40-2B (Table 1, Fig 2). Compared to 40-2A, cleavage of 40-2B in the presence of 500 nM PC is ~3-fold slower (2.2±0.4 min^-1^ for 40-2B, 6.1±0.6 min^-1^ for 40-2A), indicating that 40-2A is stimulated to a greater extent than 40-2B at this particular concentration of PC DNA.

Similar to that found in previous work, secondary site containing DNA is incapable of stimulating DNA cleavage to the same extent as primary site[5] (Table 1). At 1 μM 40-2A, the cleavage rate constant of 1 nM 40-2A is only 0.25±0.06 min^-1^, however, this rate constant is greatly increased when compared with 1 nM 40-2A (0.004±0.003 min^-1^)(Table 1). In addition, the percentage of DNA cleaved is increased from 34±12% to 78±5%.

Apart from primary and secondary site sequences, the single turnover DNA cleavage rate constants for cleavage of a non-cognate sequence, 40-NC-TA containing the sequence CTCCGGAG/CTCCGGAG, were also determined (Table 1). In the absence of any added PC DNA, the single turnover DNA cleavage rate constant (0.024±0.004 min^-1^) was ~10-fold slower than that for primary site containing DNA (40–1) under the same conditions. However, unlike the primary and secondary site sequences, the presence of 1 μM PC does not significantly stimulate cleavage of noncognate DNA (rate constant of 0.052±0.006 min^-1^, Table 1), only ~2 fold greater than without added PC DNA. In addition, the amount of the noncognate DNA cleaved is very low (<15%) in all reactions.

### Binding affinities of SgrAI for 40-2A, 40-2B, 40-NCTA

In order to determine whether the differential effects and cleavage rates for the different DNA sequences as described above resulted from different binding affinities to SgrAI, the equilibrium dissociation constants (K_D_) for the association of SgrAI with the 40 bp secondary and noncognate sequences were measured using fluorescence anisotropy (S3 Fig, Methods)(Table 2). These were measured in the presence of Ca^2+^ rather than Mg^2+^, in order to prevent cleavage of the DNA (Materials and Methods), however, because it is not cleaved by SgrAI, the K_D_ with 40-NCTA was also measured in buffer containing Mg^2+^. In the presence of Ca^2+^, K_D_ of SgrAI to secondary site DNA was considerably weaker (300–500 fold) than to primary site DNA (Table 2)[5]. Surprisingly, the K_D_ of SgrAI and the noncognate 40-NCTA was found to be only 10 fold weaker than the K_D_ of SgrAI to primary site DNA (Table 2). Further, in the presence of Mg^2+^, the K_D_ of SgrAI to the noncognate DNA was found to be approximately 5–6 fold weaker than in the presence of Ca^2+^(Table 2), but still in the low nanomolar range.

**Table 2:** Equilibrium dissociation constants of SgrAI for various DNA.

DNA	K_D_ (nM)^*a*^	Buffer ^*c*^
40–1	0.057±0.009^*b*^	LSBB with Ca^2+^
40-2A	27±2	LSBB with Ca^2+^
40-2B	17±3	LSBB with Ca^2+^
40-NCTA	0.5±0.1	LSBB with Ca^2+^
40-NCTA	2.8±0.5^*c*^	LSBB with Mg^2+^

^*a*^Values presented at the average of three independent trials ± the standard deviation.

^*b*^from Park, *et al*., 2010[8].

^*c*^buffer used in the measurements: LSBB with Ca^2+^: 20 mM Tris-OAc, 50 mM KOAc, 10 mM Ca(OAc)_2_,1 mM DTT; LSBB with Mg^2+^:20 mM Tris-OAc, 50 mM KOAc, 10 mM Mg(OAc)_2_,1 mM DTT.

### Effects of mutations in SgrAI near potential interfaces in the activated run-on oligomer

**Fig 3:**
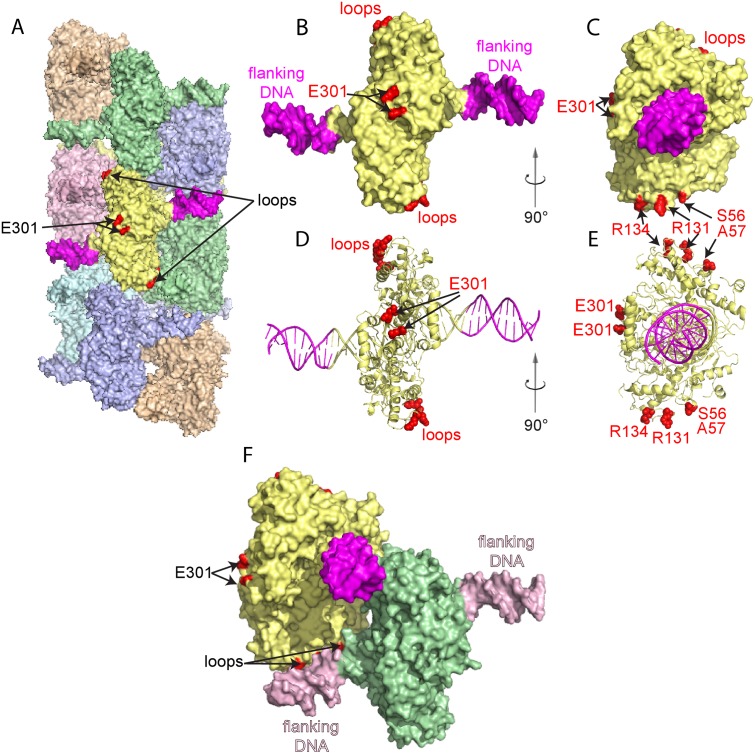
Location of additional flanking DNA and single site substitutions in SgrAI. **A**. Space filling representation of the model of the SgrAI/DNA run on oligomer derived from cryo-EM single particle reconstruction[10]. Each DNA bound SgrAI dimer is colored differently, and the outer 11 bp of flanking DNA and sites of point mutations are shown in magenta and red, respectively, in the central DNA bound SgrAI dimer [10]. E301 is located on the outer surface of SgrAI distal from the DNA binding site. **B**. Space filling model of a single DNA bound SgrAI dimer with the outer 11 bp of DNA flanking the SgrAI recognition sequence colored magenta and sites of single site substitutions (E301 and loops containing S56, S57, R131 and R134) highlighted in red. **C.** As in **B**, rotated 90°. **D** and **E.** Ribbon diagram of **B** and **C**, respectively. **F.** Close-up view of the interactions between loop residues 56–60 and 127–134 of one SgrAI chain (yellow) and the flanking DNA (light pink) bound to a neighboring SgrAI (green) in the run-on oligomer.

Single point mutations targeting the predicted interface (Fig 3) between SgrAI and the flanking DNA of neighboring SgrAI/DNA complexes in the run-on oligomer were designed using the cryo-EM model of SgrAI bound to PC DNA[10]. In this model, two loops (residues 56–60 and residues 127–134) of SgrAI closely approach the flanking DNA bound to a neighboring SgrAI (red, “loops”, Fig 3). Specifically, the mutations include S56Q, S56E, A57Q, A57E, R131A, and R134A. Purified SgrAI enzyme variant (at 1 μM) containing the single mutations was used in single turnover DNA cleavage reactions with 1 nM ^32^P labeled 18–1 and varied concentrations of unlabeled PC DNA (0, 250 and 1000 nM) as described above. Reactions were performed in triplicate, and the results given in Table 3. All of the SgrAI variants had wild type activity in cleaving 18–1 in the absence of activator PC DNA (Table 3, wild type k_obs_ is 0.094±0.015 min^-1^)[5]. However, in the presence of 250 nM and 1 μM PC DNA, the DNA cleavage rate constants differ for the enzymes with different substitutions (Table 3). Clearly, the S->Q and A->Q substitutions had little to no effect on the ability of SgrAI to become activated in DNA cleavage activity in the presence of PC DNA, with S56Q and A57Q accelerated 70 and 170 fold at 1 μM PC DNA compared to DNA cleavage rates with no added PC DNA (see Relative Acceleration, S56Q and A57Q, Table 3). The wild type SgrAI enzyme is accelerated over 200 fold under the same conditions[5] (Table 1). However, mutation of these same side chains to glutamate (S56E, A57E, Table 3) limited acceleration to only 10 and 4 fold, respectively. Similarly, the substitutions of alanine for arginine side chains at 131 and 134 also resulted in the limited acceleration of SgrAI by the presence of PC DNA (R131A, R134A, Relative Acceleration, Table 3). The binding affinities of the mutant enzymes for PC DNA were also measured (Table 4). Wild type SgrAI binds to PC DNA in the low nanomolar range (5±1 nM[5]), and the S56E, A57E, and R134A mutations did not appear to weaken this affinity (Table 4). The R131A substitution had a more measurable effect, although limited to 10 fold at the most.

**Table 3:** Single turnover DNA cleavage rate constants (k_obs_) of 1 nM ^32^P labeled 18–1 DNA, 1 μM SgrAI enzyme dimer mutant and varied concentration of unlabeled PC DNA.

Mutant	[PC DNA]	k_obs_ ^*a*^	Percent Cleaved^*a*^ ^,^ ^*b*^	Relative Acceleration^*c*^
S56Q	0	0.08±0.02	100	-
	250 nM	1.5±0.7	48±10	20
	1 μM	6±6	71±6	70
S56E	0	0.08±0.01	100	-
	250 nM	0.17±0.03	38±11	2
	1 μM	0.9±0.2	78±19	10
A57Q	0	0.09±0.01	100	-
	250 nM	3.6±0.3	72±12	40
	1 μM	15±8	80±20	170
A57E	0	0.098±0.002	100	-
	250 nM	0.27±0.03	40±20	3
	1 μM	0.39±0.06	100	4
R131A	0	0.10±0.01	100	-
	250 nM	0.5±0.1	22±19	5
	1 μM	0.28±0.02	100	3
R134A	0	0.10±0.013	100	-
	250 nM	0.9±0.4	50±30	8
	1 μM	0.8±0.2	100	8

^*a*^Values presented at the average of three independent trials ± the standard deviation.

^*b*^Percent Cleaved is the percentage of DNA cleaved by SgrAI in the assay by the given rate constant (k_obs_). In cases where less than 100% is cleaved by the given rate constant, the remainder is cleaved by a nonaccelerated rate constant.

^*c*^Relative Acceleration is the ratio of k_obs_ in the presence of 1 μM PC DNA to that in the absence of added PC DNA.

**Table 4:** Equilibrium dissociation constants of mutant SgrAI enzyme for PC DNA.

Enzyme	K_D_ (nM)^*a*^
S56E	0.26±0.02
A57E	5±2
R131A	60±20
R134A	13±6

^***a***^Values presented at the average of three independent trials ± the standard deviation.

Another investigation of the potential interfaces between SgrAI/DNA complexes in the activated, run-on oligomeric form was performed using an SgrAI variant with the substitution E301W. The single turn-over DNA cleavage rate constants of E301W SgrAI and 40–1 were determined (Table 5) in the presence of different concentrations of PC DNA and compared to those of wild type SgrAI. 1 μM E301W SgrAI cleaved the labeled 1 nM 40–1 DNA with a cleavage rate constant of 0.251±0.008 min^-1^, which is very similar to that of wild type SgrAI (0.236±0.006 min^-1^). Similarly, upon adding 1 and 2 μM PC the cleavage rate constants were enhanced to 17±2 and 16.6±0.7 min^-1^ respectively. Again, the rate constants are very similar to wild type SgrAI (16.3±1.7 and 23±2 min^-1^)(Table 1).

**Table 5:** Single-Turnover DNA Cleavage Rate Constants (k_obs_) using 1 μM E301W SgrAI and 1 nM ^32^P-labeled 40 bp primary site (40–1) DNA.

Added Unlabeled [PC]	k_obs_ ^*a*^	Percent Cleaved ^*a*^ ^,^ ^b^	Relative Acceleration^c^
0 nM	0.251±0.008	69.9±0.4	-
10 nM	0.32±0.02	76±1	1
100 nM	1.4±0.1	67.0±0.7	6
250 nM	5±1	66±1	20
500 nM	15±3	74±2	60
1 μM	17±2	76±1	70
2 μM	16.6±0.7	79.1±0.2	70

^***a***^Values presented at the average of three independent trials ± the standard deviation.

^*b*^Percent Cleaved is the percentage of DNA cleaved by SgrAI in the assay by the given rate constant (k_obs_). In cases where less than 100% is cleaved by the given rate constant, the remainder is cleaved by a nonaccelerated rate constant.

^*c*^Relative Acceleration is the ratio of k_obs_ in the presence of 1000 μM PC DNA to that in the absence of added PC DNA.

### Comparison of IM-MS CSS with those of the cryo-EM structure

Previous analyses of the SgrAI/PC DNA oligomers using ion mobility mass spectrometry (IM-MS) determined the approximate size of the oligomers in the form of a collisional cross-section (CCS) [9]. The CCS was determined for the DNA bound SgrAI dimer (DBD), as well as for oligomers containing 2, 3 and 6 DBD (S4 Fig). While these values were reported[9], a comparison with the model obtained from cryo-EM[10] was not possible at that time. S4 Fig now compares the calculated CCS from the cryo-EM model (open circles, S4 Fig) with the experimentally determined CCS using IM-MS (filled circles, S4 Fig).

## Discussion

Our current working model for the activation of DNA cleavage by SgrAI involves the assembly of SgrAI/DNA complexes into a run-on oligomer that stabilizes an activated conformation of the SgrAI enzyme [10]. This activated conformation has an accelerated rate of DNA cleavage of both primary and secondary site sequences as compared to the low activity, dimeric form of SgrAI. The oligomer is a “run-on” oligomer due to its ability to add additional SgrAI/DNA complexes at one or both ends of the oligomer (a left handed helix) in a potentially unending manner (Fig 3A). The model predicts that formation of the run-on oligomer is favored only when SgrAI is bound to its primary recognition sequence, but also that the run-on oligomer is capable of incorporating, and thereby activating, SgrAI bound to secondary site sequences. This model for SgrAI activation has been derived from several previous studies of DNA cleavage by SgrAI [5,6,24], as well as structural studies including high resolution structures of the low activity form [8,11,12] and an 8.6 Å cryo-EM structure of the activated form (the run-on oligomer)[10]. Herein we sought to both test predications made based on the cryo-EM model using site directed mutagenesis, as well as to expand our understanding of various other aspects of SgrAI activation (see below).

Our standard assay to investigate the activation of SgrAI utilizes a low concentration of reporter DNA containing the SgrAI recognition site (^32^P labeled), high concentrations of the SgrAI enzyme, and varied concentrations of unlabeled activator DNA. Typically the activator DNA is a cleaved version of the primary site sequence (i.e. PC DNA, Fig 1), however, we have also tested other versions of the primary site including the intact primary site sequence (40–1, Fig 1) and an uncleavable version of the intact primary site sequence (22-1-3’S, Fig 1), as well as a secondary site sequence (40-2A, Fig 1). The single turnover DNA cleavage conditions (i.e. the large excess of SgrAI concentration over reporter DNA concentration) allows for the measurement of a first order rate constant for DNA cleavage (k_obs_, Table 1). We find that this rate constant varies with the concentration of activator DNA (“Added Unlabeled Activator DNA”, Table 1), becoming faster with higher concentrations of activator DNA (see “Relative Acceleration”, Table 1). Previously we have discussed our interpretation of this phenomenon, namely in capturing in part or in whole, the rate of run-on oligomer formation (which would depend on the concentration of SgrAI bound to activator DNA) in the k_obs_ [5]. In the current study, we have filled in several missing aspects of the SgrAI activation model. First, we have found that use of a reporter DNA molecule with a greater number of base pairs flanking the SgrAI recognition site (i.e. 40–1 vs. 18–1, Fig 1) increases the relative acceleration of DNA cleavage at intermediate concentrations of activator DNA (compare at 100 nM PC DNA, Table 1, Fig 2). Previously we have shown that the flanking DNA was important in the *activator* DNA, where a greater number of flanking base pairs results in a greater degree of SgrAI activation[10]. In either case, whether considering the reporter or the activator DNA, these results are readily explained by our model of the activated form of SgrAI; in the cryo-EM model of the run-on oligomer, the residues from one SgrAI/DNA complex appear close enough to make ionic, hydrogen bonding, and/or van der Waals interactions with flanking DNA (magenta, Fig 3A) of the neighboring SgrAI/DNA complex (Fig 3A and 3F). In the DNA cleavage reactions, the run-on oligomer would be composed largely of SgrAI bound to PC DNA stabilized by interactions between the flanking DNA from one SgrAI/PC DNA complex to SgrAI residues of neighboring SgrAI/PC DNA complex (as well as by protein-protein interactions between neighboring SgrAI). Similarly, the SgrAI bound to the reporter DNA (i.e. 18–1 or 40–1) would be activated by being assimilated into this run-on oligomer and making the same types of interactions. The flanking DNA highlighted in magenta (Fig 3A, and pink and magenta, Fig 3F) are present in 40–1, but not 18–1. Therefore, SgrAI bound to 40–1 would be expected to bind with a higher affinity to the run-on oligomer and therefore be activated at lower concentrations of the run-on oligomer.

Similarly, measurements with a secondary site containing more flanking base pairs (40-2A vs. 18–2, Fig 1) result in considerably greater degrees of activation (up to 7000 fold in Relative Acceleration, Table 1)(compare 40-2A and 18–2, Fig 2), however, the measured rate constants for DNA cleavage of the secondary site at the highest concentration of activator DNA tested still fall short of those measured for the cleavage of primary site (compare 40-2A and 40-2B with 40–1 and 18–1 at 1000 nM PC DNA, Fig 2). The dependence of the rate constant of DNA cleavage on the concentration of activator DNA also differs between the secondary site (40-2A and 40-2B, Fig 2) and the primary site with the same number of flanking base pairs (40–1, Fig 2), requiring more activator DNA to achieve similar levels of activation for the cleavage of the secondary site (Fig 2). This cannot merely be explained by a lower affinity of SgrAI for the secondary site, since measured affinities were found to be in the 10–30 nM range (note: these were measured in the presence of Ca^2+^, but measurements with the catalytically relevant Mg^2+^ indicate only a 5–6 fold weaker K_D_, Table 2). Instead we interpret this result as a lowered affinity of the run-on oligomer to the complex containing SgrAI bound to secondary site. This is consistent with our model for the inability of secondary site DNA to activate SgrAI, namely that binding to the secondary site by SgrAI favors the low activity conformation which would have a lower affinity for the run-on oligomer, since the run-on oligomer preferentially binds (and thereby stabilizes) the activated conformation [10].

The results with single mutations in SgrAI, designed to disrupt the run-on oligomer without disrupting SgrAI structure or DNA binding, also support our model for SgrAI activation (Tables 3–5, Fig 3). These substitutions were located in either of two loops making close contact to the flanking DNA of a neighboring SgrAI/DNA complex in the run-on oligomer structure (Fig 3A and 3F), or in one case, distal from any contacts (E301W, Fig 3A and Fig 3F). The substitutions with the greatest effect were those introducing a negative charge (S56E, A57E, Table 3), or those removing a positive charge (R131A and R134A, Table 3), consistent with charge repulsion or alternatively loss of charge attraction to DNA. However, these substitutions did not significantly affect DNA binding by SgrAI (Table 4) or the basal rate of DNA cleavage by SgrAI in the absence of activator DNA (compare 0 nM concentration of PC DNA, Table 3, to that of wild type SgrAI with ^32^P-labeled 18–1, Table 1), indicating minimal disruption of the low activity form (i.e. the dimeric DNA bound SgrAI). The results are therefore consistent with the perturbation of interactions between flanking DNA (magenta, Fig 3A, pink and magenta, Fig 3F) and neighboring SgrAI in the run-on oligomer. In contrast, the E301W substituted SgrAI behaved as wild type in the DNA cleavage assays (Table 5), consistent with the position of E301 distal from any interfaces in the SgrAI/DNA complex or in the run-on oligomer (Fig 3). Finally, the last piece we present here supporting our run-on oligomer model of SgrAI derives from two previous independent structural investigations, one using mass spectrometry (ion mobility mass spectrometry[9]) and the other the cryo-EM structural study[10], which both provide consistent measures of the size of the run-on oligomer containing 1–6 copies of the DNA bound SgrAI dimer (DBD) (S4 Fig).

In addition to the studies probing the structural model of the run-on oligomer described above, we present the results of several new studies probing the parameters of SgrAI activation. First, we tested whether the uncleaved primary site could activate SgrAI to the same extent as the cleaved. Comparing 1 μM 40–1 to 2 μM PC DNA (since each SgrAI dimer binds one 40–1 or two PC DNA [9]), we found that PC DNA appears to activate SgrAI to twice the value of the observed rate constant (using 40–1 as the reporter DNA, Table 1). Since the uncleaved DNA could in principle be cleaved by SgrAI in these assays, we also tested the allosteric activating properties of an uncleavable version of the primary site (22-1-3’S, Fig 1), and found it capable of activating SgrAI by 16 fold (Table 1). Therefore, uncleaved primary site is in fact capable of activating SgrAI, however, the cleaved version may actually provide more robust activation.

Regarding the two types of SgrAI secondary site, those with the substitution in the 2^nd^/7^th^ nucleotide position (i.e. CRCCGGGG) and those with the substitution in the 1^st^/8^th^ position (i.e. CRCCGGY(A / C / T))(the primary site sequence is CRCCGGYG), we found qualitative and quantitative differences in cleavage by SgrAI (40-2A is of the first type, 40-2B is of the second type, Figs 1–2, Table 1). First, cleavage of the first type requires lower concentrations of activator DNA to accelerate its cleavage by SgrAI (Fig 2). Second, in the absence of activation the cleavage of the second type (40-2B, Table 1) is much slower in one strand than the other (slower in the bottom strand, Table 1). This may be because on this strand the cleavage site is closer to the substitution in the sequence (the difference in the sequence compared to the primary site sequence, Fig 1), hence potentially resulting in greater structural disruption of the cleavage active site, a phenomenon we have seen before with a different restriction endonuclease [25,26]. It is interesting that the differences in the rates of cleavage of the two strands disappear with higher concentrations of activator DNA (Table 1). This suggests that an asymmetry in the low activity conformation is absent in the activated conformation. The mechanism for this change awaits the high resolution structures of SgrAI (activated and low activity forms) bound to this type of secondary site DNA; to date all structures of SgrAI bound to the secondary site have been with the first type, and in the low activity conformation [12], and the atomic details of the structure of the activated state of SgrAI is limited by the resolution cryo-EM study (8.6 A) and is with primary site DNA[10].

Finally, we investigated the potential for activated cleavage of a noncognate sequence, one that differs from primary at two base pairs, and is essentially a symmetrized version of a secondary site (i.e. CTCCGGAG, 40-NCTA, Fig 1). We found that SgrAI cleaved only a small percentage of this DNA, and with very slow k_obs_ (Table 1). Unlike with secondary site DNA, the activator DNA was not able to activate SgrAI to cleave this sequence (Table 1). The lack of cleavage was not due to lack of binding to this sequence, as the affinity for the DNA was measured to be in the low nanomolar range, with either Ca^2+^ or Mg^2+^ (Table 2). This result suggests that the ability of SgrAI to cleave secondary site sequences is not merely loss of recognition to the outer two base pairs, otherwise 40-NCTA should also be cleaved by SgrAI. We suggest that binding to the noncognate DNA may result in blocking SgrAI from attaining the activated conformation, and therefore from binding to the run-on oligomer.

The unusual mechanism of activation and modulation of substrate specificity exhibited by SgrAI may actually be shared to a greater or lesser extent by a growing list of enzymes. Run-on filament formation that affects enzyme activity has been shown by a handful of other enzymes, including IRE1[2] (a kinase/RNase involved in the unfolded protein response), acetyl-CoA carboxylase [3] (ACC), CTP synthase [4,27,28] (CTPS), and RIP1/RIP3 kinases [29] (involved in programmed necrosis). The growing body of knowledge regarding the mechanisms of these enzymes allows for a comparison of mechanistic details with those of SgrAI. First, SgrAI is activated from a low activity state to one that is 200–1000 fold more active in the run-on oligomer; IRE1, ACC and RIP1/RIP3 are also activated in their oligomeric/filamentous forms, while CTPS is inhibited. The actual degree of activation can vary in those activated by oligomerization, being 200–1000 fold for SgrAI, 60 fold for ACC [3], and over 100,000 fold in the case of the RNase activity of IRE1 [2] (that for RIP1/RIP3 has not been quantitated). Filament formation is stimulated by binding of substrate only in the case of SgrAI, while IRE1, ACC, RIP1/RIP3, and CTPS form filaments in response to binding to activators (unfolded proteins in the case of IRE1, and the allosteric effector citrate in the case of ACC), products (CTP, which is the product of CTPS), or phosphorylation (RIP1/RIP3). Phosphorylation also appears to be involved in further oligomerization of IRE1, once initial oligomerization has begun [2,7]. In addition, the substrates of CTPS can induce its depolymerization to produce the active form of the enzyme [27]. Modulation of substrate specificity upon run-on oligomerization or filament formation also occurs in the SgrAI system. Only IRE1 appears to display a similar property, as it cleaves other RNA molecules in addition to its target mRNA when in its longest filaments [7]. Some apparently unique features in the SgrAI system include the fact that it binds tightly to both types of sites, primary and secondary, in its low activity form but only cleaves primary in that state (secondary site DNA is cleaved significantly by SgrAI only in the run-on oligomer). In addition, both the substrate (uncleaved primary) and product (cleaved primary DNA) stabilize the run-on oligomeric form of SgrAI. Another characteristic that differentiates these systems is the potential for rapid association and dissociation kinetics, which are likely in all cases except the RIP1/RIP3 system, which appears to form an irreversible amyloid [29]. Finally, the proposed biological role for run-on oligomer/filament formation includes rapid activation (or deactivation in the case of CTPS) in all cases, and increased substrate binding (through a larger interface in the case of IRE1 and also possibly RIP1/RIP3). In the case of CTPS, the filament has been proposed to be a further mechanism of fine tuning the enzyme’s function to the environmental concentrations of substrate and product by creating a readily activatable pool of inactive enzymes [27]. Only the run-on oligomer formed by SgrAI has the proposed role of sequestration of activated enzymes[5,10]. We postulate that this mechanism evolved due to the relatively long genome of *Streptomyces griseus*, from which SgrAI derives; the longer genome would result in a greater potential number of DNA cleavage sites, which must be protected by the cognate methyltransferase to prevent damage to the host DNA by the SgrAI endonuclease. If the activity of the methyltransferase is limited, perhaps due to cofactor availability, the system could respond evolutionarily by reducing the activity of the endonuclease. The unusually long recognition sequence of SgrAI (8 bp vs. 4–6 bp), and low DNA cleavage activity in the absence of activation both reduce SgrAI activity, but also concurrently diminish the enzyme’s effectiveness against invading phage DNA. However, the ability of SgrAI to both activate its cleavage activity more than 200 fold, and also expand its sequence specificity (from 3 to 17 different sequences) in the presence of unmethylated primary sites (e.g. as expected in phage DNA) recovers its activity against phage. Run-on oligomer formation may have evolved to sequester activated SgrAI on the phage and away from the host DNA, since cleavage at presumably unmethylated secondary sites in the host DNA would be damaging to the host. In addition, the run-on oligomer could also serve to rapidly activate many SgrAI by assimilating a potentially unlimited number of DNA bound SgrAI into a growing oligomer and/or to block replication and transcription of the phage DNA. In conclusion, SgrAI appears to be a member of a small (but perhaps growing [30–34]) group of enzymes known to be modulated by the formation of run-on oligomers or filaments. Yet, SgrAI maintains several unique characteristics as well. Further work will be needed to determine how generalizable the mechanisms of SgrAI and the other similarly behaving enzymes are in nature.

## Supporting Information

S1 Fig Phosphorothiolate substituted DNA is not cleaved by SgrAI. **A.** 1 nM ^32^P labeled 22–1 (left) or 22-1-3’P (right) and 1 μM SgrAI in kinetic buffer quenched following varied times after mixing. DNA was resolved on denaturing PAGE and visualized via autoradiography. UC = uncleaved 22mer DNA, C = cleaved DNA, NS = products of nonspecific cleavage likely from contaminating nucleases. **B.** Side-by-side comparison of late time-points from **A** showing that the products from nonspecific cleavage (NS), which appear in reactions with either DNA, are running faster than specific cleavage products (C). UC = uncleaved 22mer. 1 = 22–1, 2 = 22-1-3’S. **C.** 1 nM ^32^P labeled 22-1-3’S (left) or 22–1 (right) with 1 μM SgrAI and 1 μM unlabeled PC DNA, quenched following different times of incubation after mixing and resolved using denaturing PAGE and autoradiography. Labels as in **A**. **D.** 1 nM ^32^P labeled 22–1 (left) or 22-1-3’s (right) with 1 μM SgrAI and 1 μM unlabeled 22–1 (left) or 22-1-3’S (right), quenched following different times of incubation after mixing and resolved using denaturing PAGE and autoradiography. Labels as in **A**.
(DOCX)

S2 Fig Accelerated DNA cleavage in the presence of phosphorothiolate substituted DNA. **A.** Example of an autoradiogram of a denaturing gel analyzing the 1 nM ^32^P-18-1 after different incubation times with 1 μM SgrAI (see Methods for single turnover DNA cleavage reactions). **B.** Example of analysis of data from A (filled circles), with fit to single exponential function (see Methods) giving a rate constant (k_obs_) of 1.36 min^-1^ and R of 0.99667.
(DOCX)

S3 Fig Titration of fluorescein labeled noncognate DNA Flo-40-NCTA with SgrAI, using fluorescence anisotropy to detect the formation of the protein-DNA complex. Experimentally measured data is shown as solid circles, and the fit to a 1:1 binding model (see Methods) shown as a solid line giving a K_D_ of 1.1 nM with an R value of 0.99848.
(DOCX)

S4 Fig Comparison of experimentally determined and predicted collisional cross sections of SgrAI/DNA run-on oligomers. Collisional cross-sections (CCS) of SgrAI/DNA complexes determined using ion mobility mass spectrometry (IM-MS, filled circles) and those calculated using the oligomer model from the cryo-EM analysis, and the scaled PA method (Materials and Methods)(open circles). The line is the best linear fit to the IM-MS CCS. Inset is a cartoon representation of the cryo-EM model with six DBD (DNA omitted for clarity).
(DOCX)

S1 Methods Mutagenesis. (DOCX)

S2 Methods Protein Purification. (DOCX)

S1 Results Phosphorothiolate substituted DNA is not cleaved by SgrAI. (DOCX)
