# Supplementary material for: Probing the Run-On Oligomer of Activated SgrAI Bound to DNA
Source: PLoS One. 2015 Apr 16;10(4):e0124783. doi: 10.1371/journal.pone.0124783 (PMC4399878; doi:10.1371/journal.pone.0124783)
Supplement: S1 Fig — A. 1 nM 32P labeled 22–1 (left) or 22-1-3’P (right) and 1 μM SgrAI in kinetic buffer quenched following varied times after mixing. DNA was resolved on denaturing PAGE and visualized via autoradiography. UC = uncleaved 22mer DNA, C = cleaved DNA, NS = products of nonspecific cleavage likely from contaminating nucleases. B. Side-by-side comparison of late time-points from A showing that the products from nonspecific cleavage (NS), which appear in reactions with either DNA, are running faster than specific cleavage products (C). UC = uncleaved 22mer. 1 = 22–1, 2 = 22-1-3’S. C. 1 nM 32P labeled 22-1-3’S (left) or 22–1 (right) with 1 μM SgrAI and 1 μM unlabeled PC DNA, quenched following different times of incubation after mixing and resolved using denaturing PAGE and autoradiography. Labels as in A. D. 1 nM 32P labeled 22–1 (left) or 22-1-3’s (right) with 1 μM SgrAI and 1 μM unlabeled 22–1 (left) or 22-1-3’S (right), quenched following different times of incubation after mixing and resolved using denaturing PAGE and autoradiography. Labels as in A. (DOCX) [file pone.0124783.s001.docx]

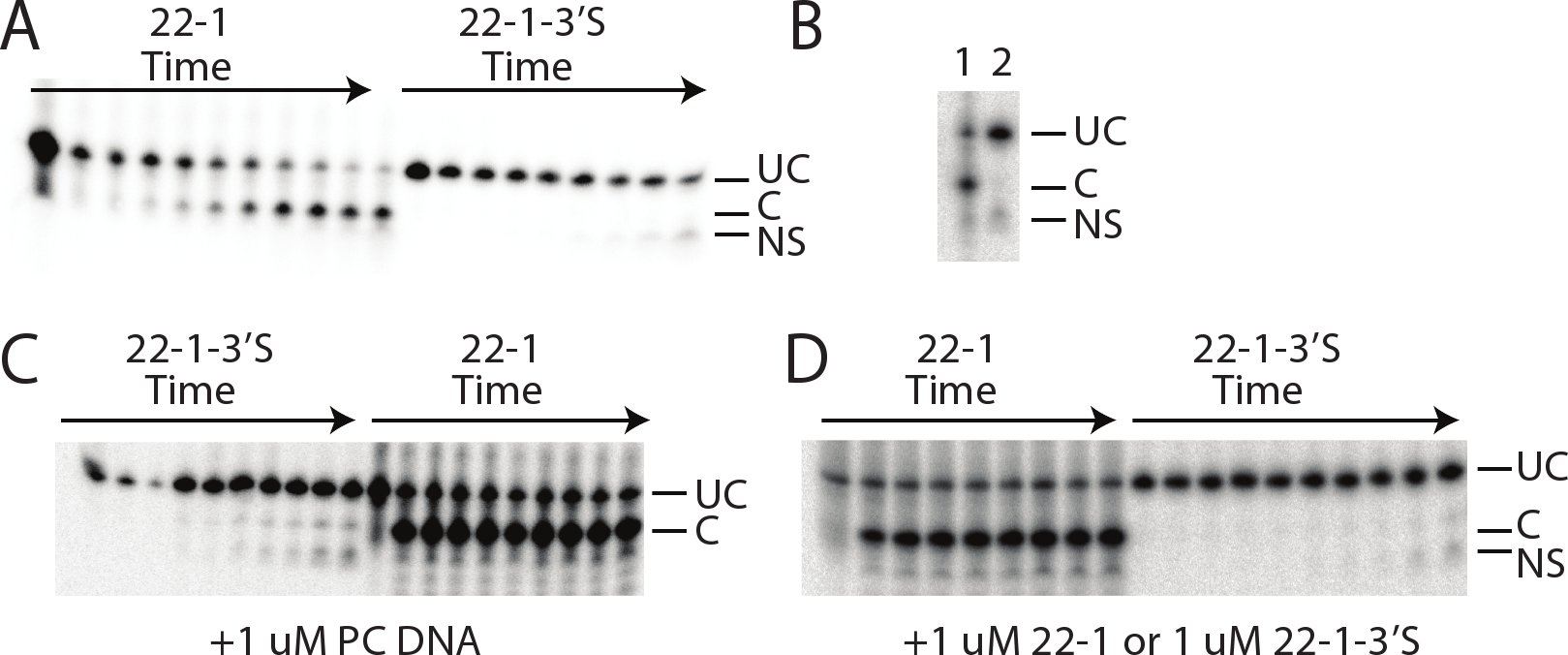


**S1 Figure. Phosphorothiolate substituted DNA is not cleaved by SgrAI**. **A.** 1 nM ^32^P labeled 22-1 (left) or 22-1-3’P (right) and 1 μM SgrAI in kinetic buffer quenched following varied times after mixing. DNA was resolved on denaturing PAGE and visualized via autoradiography. UC=uncleaved 22mer DNA, C=cleaved DNA, NS=products of nonspecific cleavage likely from contaminating nucleases. **B.** Side-by-side comparison of late time-points from **A** showing that the products from nonspecific cleavage (NS), which appear in reactions with either DNA, are running faster than specific cleavage products (C). UC=uncleaved 22mer. 1=22-1, 2=22-1-3’S. **C.** 1 nM ^32^P labeled 22-1-3’S (left) or 22-1 (right) with 1 μM SgrAI and 1 μM unlabeled PC DNA, quenched following different times of incubation after mixing and resolved using denaturing PAGE and autoradiography. Labels as in **A**. **D.** 1 nM ^32^P labeled 22-1 (left) or 22-1-3’s (right) with 1 μM SgrAI and 1 μM unlabeled 22-1 (left) or 22-1-3’S (right), quenched following different times of incubation after mixing and resolved using denaturing PAGE and autoradiography. Labels as in **A**.
