# Supplementary material for: Probing the Run-On Oligomer of Activated SgrAI Bound to DNA
Source: PLoS One. 2015 Apr 16;10(4):e0124783. doi: 10.1371/journal.pone.0124783 (PMC4399878; doi:10.1371/journal.pone.0124783)
Supplement: S2 Fig — A. Example of an autoradiogram of a denaturing gel analyzing the 1 nM 32P-18-1 after different incubation times with 1 μM SgrAI (see Methods for single turnover DNA cleavage reactions). B. Example of analysis of data from A (filled circles), with fit to single exponential function (see Methods) giving a rate constant (kobs) of 1.36 min-1 and R of 0.99667. (DOCX) [file pone.0124783.s002.docx]

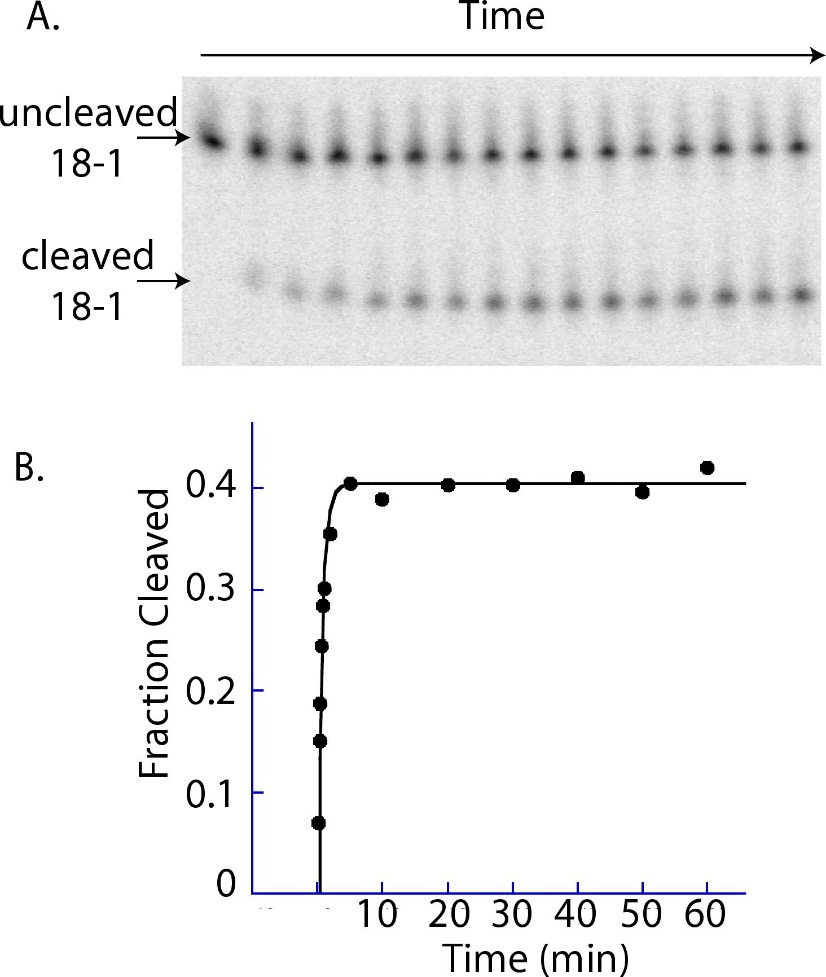


**S2 Figure.** **Accelerated DNA cleavage in the presence of phosphorothiolate substituted DNA.** **A.** Example of an autoradiogram of a denaturing gel analyzing the 1 nM ^32^P-18-1 after different incubation times with 1 μM SgrAI (see Methods for single turnover DNA cleavage reactions). **B.** Example of analysis of data from A (filled circles), with fit to single exponential function (see Methods) giving a rate constant (k_obs_) of 1.36 min^-1^ and R of 0.99667.
