# Supplementary material for: Probing the Run-On Oligomer of Activated SgrAI Bound to DNA
Source: PLoS One. 2015 Apr 16;10(4):e0124783. doi: 10.1371/journal.pone.0124783 (PMC4399878; doi:10.1371/journal.pone.0124783)
Supplement: S3 Fig — Experimentally measured data is shown as solid circles, and the fit to a 1:1 binding model (see Methods) shown as a solid line giving a KD of 1.1 nM with an R value of 0.99848. (DOCX) [file pone.0124783.s003.docx]

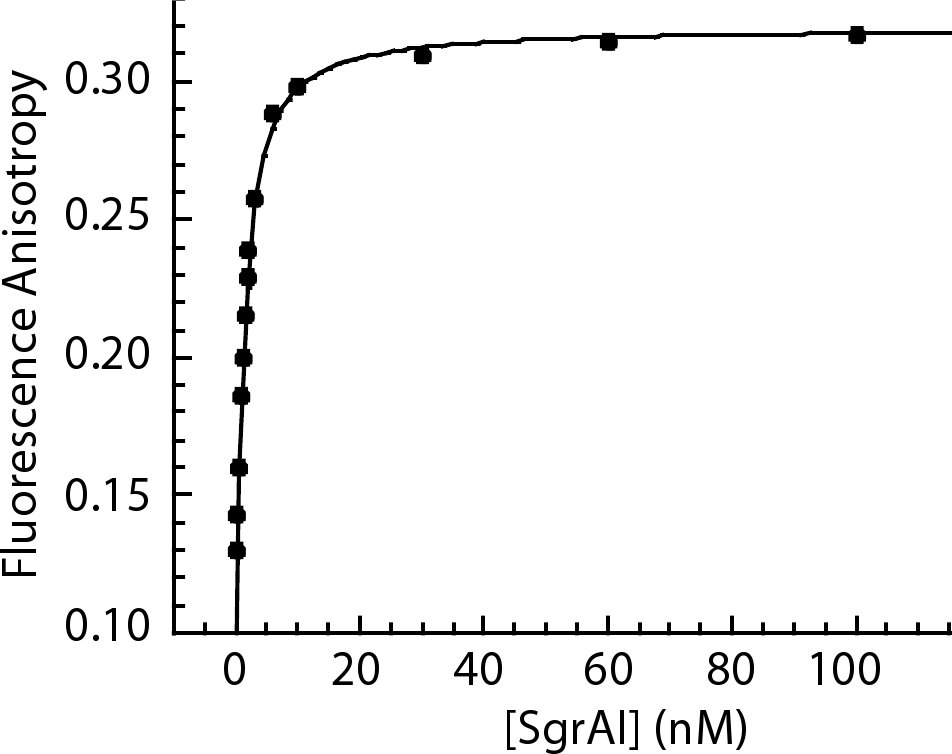


**S3 Figure.** . **Titration of fluorescein labeled noncognate DNA Flo-40-NCTA with SgrAI, using fluorescence anisotropy to detect the formation of the protein-DNA complex.** Experimentally measured data is shown as solid circles, and the fit to a 1:1 binding model (see Methods) shown as a solid line giving a K_D_ of 1.1 nM with an R value of 0.99848.
