# Supplementary material for: Probing the Run-On Oligomer of Activated SgrAI Bound to DNA
Source: PLoS One. 2015 Apr 16;10(4):e0124783. doi: 10.1371/journal.pone.0124783 (PMC4399878; doi:10.1371/journal.pone.0124783)
Supplement: S4 Fig — Collisional cross-sections (CCS) of SgrAI/DNA complexes determined using ion mobility mass spectrometry (IM-MS, filled circles) and those calculated using the oligomer model from the cryo-EM analysis, and the scaled PA method (Materials and Methods)(open circles). The line is the best linear fit to the IM-MS CCS. Inset is a cartoon representation of the cryo-EM model with six DBD (DNA omitted for clarity). (DOCX) [file pone.0124783.s004.docx]

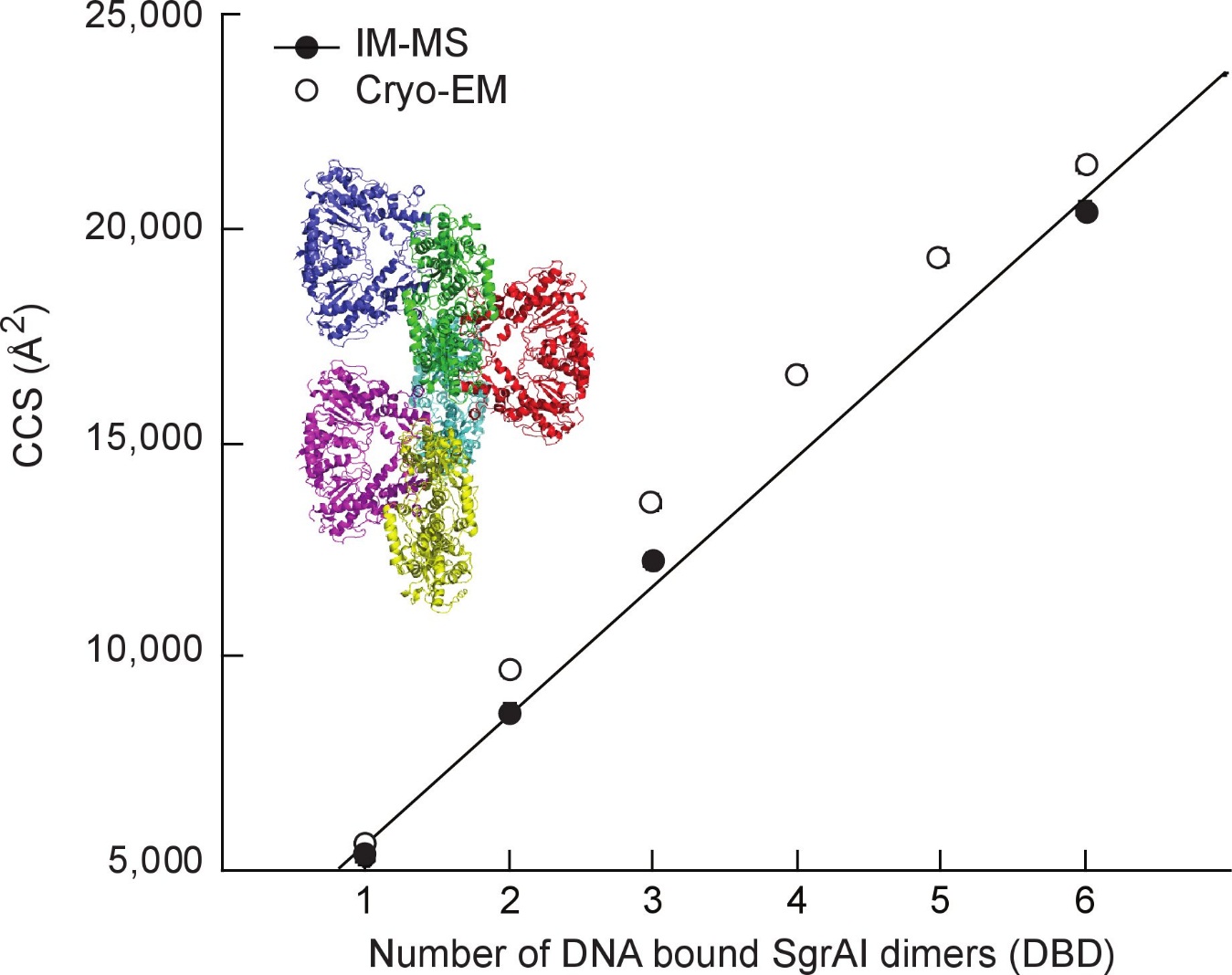


**S4 Figure.** **Comparison of experimentally determined and predicted collisional cross sections of SgrAI/DNA run-on oligomers.** Collisional cross-sections (CCS) of SgrAI/DNA complexes determined using ion mobility mass spectrometry (IM-MS, filled circles) and those calculated using the oligomer model from the cryo-EM analysis, and the scaled PA method (Materials and Methods)(open circles). The line is the best linear fit to the IM-MS CCS. Inset is a cartoon representation of the cryo-EM model with six DBD (DNA omitted for clarity).
