# Supplementary material for: Probing the Run-On Oligomer of Activated SgrAI Bound to DNA
Source: PLoS One. 2015 Apr 16;10(4):e0124783. doi: 10.1371/journal.pone.0124783 (PMC4399878; doi:10.1371/journal.pone.0124783)
Supplement: S1 Methods — (DOCX) [file pone.0124783.s005.docx]

**S1 Methods. Mutagenesis**

The E301W substitution in SgrAI was prepared using a mutagenesis protocol described previously[1]. Q5^®^ Site Directed Mutagenesis Kit (New England Biolabs, Ipswich, MA, USA) was used to add a His-Tag sequence (six histidine residues) to the 3'-end of the *sgrAI* gene and to introduce the amino acid substitutions at positions coding for Ser56 and Ala57. The original pET21a_SgrAI plasmid[2] possesses a 6xHis coding sequence, but it is separated from the *sgrAI* gene by the 13-nt insert. In the first mutagenesis round, we deleted these 13 nucleotides (positions 158-170 nt) by designing the primers that flank the region to be deleted: 5’-CCACCACCACTGAGATCCGGCT (sgr-hisFw) and 5’-TGGTGGTGGCCGTTGACCAGCTCCATGCGT (sgr-hisRev). PCR amplification of the entire pET21a_SgrAI plasmid and a KLD reaction of the site-directed mutagenesis were performed following the instructions recommended by the manual of the Q5^®^ Site Directed Mutagenesis Kit. The new construct, pET21a_SgrAI_6his was then used as a template in the second mutagenesis round to introduce the desired mutations into the *sgrAI* gene. To substitute Ser56 or Ala57 of SgrAI with either Glu or Gln, four mutagenic primers were designed where the codon TCA or GCT was replaced by either codon GAA or by codon CAG respectively:

5’-TGGATTCGCTGAAGCTGGGATTAACGGA (S56E-Fw),

5’-TGGATTCGCTCAGGCTGGGATTAACGGA (S56Q-Fw),

5’-ATTCGCTTCAGAAGGGATTAACGGAATG (A57E-Fw) and

5’- ATTCGCTTCACAGGGGATTAACGGAATG (A57Q-Fw).

Four PCR amplification reactions were performed, each reaction using one of the above primers and the respective reverse primer, either 5’-GGGATTACCTCACCGAAG (S56-Rev) or 5’-CCAGGGATTACCTCACCG (A57-Rev). The obtained PCR products were further used to set up four KLD reactions of the Q5^®^ Site Directed Mutagenesis Kit. Plasmid DNA was purified from 4 individual recombinant colonies representing each mutagenesis reaction. To confirm nucleotide sequence the plasmids were sequenced across the *sgrAIR* gene. No sequence changes, except for the anticipated codon changes (S56E, S56Q, A57E or A57Q), were observed.

The C terminal his-tag on wtSgrAI-his was created by utilizing the pET21a_SgrAI vector harboring the *sgrAI* gene as a template in a PCR with primers 5’ TAGCATGACTGGTGGACA and 5’ GCCGTTCACCAGCTCC. 25 ng of the amplicon was used in another PCR with primers 5’ TAGCATGACTGGTGGACA and 5’TGCATCTCGAGTGCTCATGACTCATCAGTGGTGGTGGTGGTGGTGGCCGTTCACCAGCTCC, which introduced a 6X His tag to the C-terminus of the *sgrAI* gene. The resulting amplicon DNA was digested and ligated into the pET21a_SgrAI plasmid [2] using the BamHI and XhoI sites. DNA sequencing confirmed the sequence of the wtSgrAI-his.

Preparation of the R131A and R134A SgrAI coding sequences utilized the primer overlap extension method [3]. The following PCR primers were used in two separate PCR reactions with the upstream 5’TAGCATGACTGGTGGACA and downstream 5’TTTCGGGCTTTGTTAGCAG primers: R131A; 5’TGGCGCTCTCAGCCGgcgTACAGTAGGCCGACG (with the upstream primer) and 5’CGTCGGCCTACTGTAcgcCGGCTGAGAGCGCCA (with the downstream primer), and R134A; 5’CAGCCGCGGTACAGTgcgCCGACGCTGAGTCCG (with the upstream primer) and 5’CGGACTCAGCGTCGGcgcACTGTACCGCGGCTG (with the downstream primer). A third PCR reaction was performed with the products of the upstream and downstream amplicons, and the primers 5’TAGCATGACTGGTGGACA and 5’TTTCGGGCTTTGTTAGCAG. The amplicon inserts were ligated into the pET21a_SgrAI vector under the same conditions as the cloning of the wtSgrAI-his tag.

**References cited**

1. Park CK, Joshi HK, Agrawal A, Ghare MI, Little EJ, et al. (2010) Domain swapping in allosteric modulation of DNA specificity. PLoS Biol 8: e1000554.

2. Dunten PW, Little EJ, Gregory MT, Manohar VM, Dalton M, et al. (2008) The structure of SgrAI bound to DNA; recognition of an 8 base pair target. Nucleic Acids Res 36: 5405-5416.

3. Aiyar A, Xiang Y, Leis J (1996) Site-directed mutagenesis using overlap extension PCR. Methods Mol Biol 57: 177-191.
