# Supplementary material for: Probing the Run-On Oligomer of Activated SgrAI Bound to DNA
Source: PLoS One. 2015 Apr 16;10(4):e0124783. doi: 10.1371/journal.pone.0124783 (PMC4399878; doi:10.1371/journal.pone.0124783)
Supplement: S2 Methods — (DOCX) [file pone.0124783.s006.docx]

**S2 Methods. Protein Purification**

Wild type (without his-tag) and E301W SgrAI were prepared as described [1] with some modifications. Briefly, pET21a_SgrAIR (containing either WT or E301W) plasmid was transformed into T7 Express Competent *E. coli* cells (High Efficiency, New England Biolabs, Ipswich, MA, USA), which constitutively express the MspI.M methyltransferase to protect the host genome. The cells were grown in five batches of 18 L cultures at 37**°**C until an OD_600_ of 0.5. The cells were then induced with 0.4 mM IPTG and left to grow overnight at 17**°**C. The cells were then harvested by centriguation at 5000 rpm for 30 minutes, frozen in liquid nitrogen and stored at -80**°**C. The frozen cell pellets were resuspended in breaking buffer (100 mM Tris-HCl (pH 8.0@RT), 800 mM NaCl, 10 mM EDTA, 10 mM 2-mercaptoethanol, and 0.1 mM PMSF) and sonicated using Branson Sonifier 450 (Branson, Danbury, CT, USA). The lysed cells were centrifuged for 1 hour at 9,000 rpm, followed by centrifugation at 40,000 rpm for 1 hour in a L8-70M Ultracentrifuge (Beckman Coulter, Indianapolis, IN, USA). The enzyme was purified using FPLC (GE Healthcare Biosciences, Pittsburgh, PA, USA) chromatography and the following chromatographic resins: Heparin FF Sepharose, SP FF Sepharose, Q FF Sepharose, followed by a second Heparin FF Sepharose chromatographic step. Finally, SgrAI enzyme was dialyzed into storage buffer (20 mM Tris-HOAc (pH 8.0@RT), 50 mM KOAc, 0.1 mM EDTA, 1 mM DTT, 50% glycerol), aliquoted into single use aliquots, flash frozen in liquid nitrogen, and stored at -80°C.

The his-tagged proteins were expressed in BL21(DE3) *E. coli* containing the pLysS plasmid (Millipore, Inc., Billerica, MA, USA.) and the Msp.M expression plasmid[1], and were purified using TALON metal affinity resin (Clonetech, Inc., Mountain View, CA, USA). In the case wtSgrAI-his, as well as enzymes containing the S56E, S56Q, A57E, and A57Q substitutions, a heparin chromatographic step was also utilized. First, the cell lysate was incubated with TALON resin in lysis buffer (50 mM sodium phosphate buffer (pH 8.0@RT), 800 mM NaCl, 10 mM imidazole, and 1 mM 2-mercaptoethanol) 30 min to overnight. The unbound cell lysate was washed away using wash buffer (50 mM sodium phosphate buffer (pH 8.0@RT), 300 mM NaCl, 20 mM imidazole, and 1 mM 2-mercaptoethanol) followed by high salt wash buffer (50 mM sodium phosphate buffer (pH 8.0@RT), 2 M NaCl, 20 mM imidazole, and 1 mM 2-mercaptoethanol). Finally, the protein was eluted using elution buffer (50 mM sodium phosphate buffer (pH 8.0@RT), 300 mM NaCl, 250 mM imidazole, and 1 mM 2-mercaptoethanol). In the case of wild type, S56E, S56Q, A57E, and A57Q SgrAI enzymes, partially purified protein was then excessively dialyzed into Heparin A buffer (50 mM Tris-HCl (pH 8.0@RT), 50 mM NaCl, 0.1 mM EDTA, 10 mM 2-mercaptoethanol). The dialyzed protein was purified using Heparin FF chromatography (GE Healthcare Biosciences, Pittsburgh, PA, USA) and a gradient of Heparin B buffer (50 mM Tris-HCl (pH 8.0@RT), 1 M NaCl, 0.1 mM EDTA, 10 mM 2-mercaptoethanol). Purity of the protein was confirmed using SDS-PAGE. The purified protein was then aliquoted into single use aliquots, flash frozen in liquid nitrogen, and stored at -80°C.

**References cited**

1. Dunten PW, Little EJ, Gregory MT, Manohar VM, Dalton M, et al. (2008) The structure of SgrAI bound to DNA; recognition of an 8 base pair target. Nucleic Acids Res 36: 5405-5416.
