# Supplementary material for: Probing the Run-On Oligomer of Activated SgrAI Bound to DNA
Source: PLoS One. 2015 Apr 16;10(4):e0124783. doi: 10.1371/journal.pone.0124783 (PMC4399878; doi:10.1371/journal.pone.0124783)
Supplement: S1 Results — (DOCX) [file pone.0124783.s007.docx]

**S1 Results. Phosphorothiolate substituted DNA is not cleaved by SgrAI**

Figure S1 shows the autoradiograms of denaturing gels examining cleavage of ^32^P labeled DNA following incubation with SgrAI in single turnover DNA cleavage assays (1 μM SgrAI, 1 nM ^32^P labeled DNA, 1 μM unlabeled activator DNA, 37ºC, in 20 mM Tris-HOAc (pH 8.0@RT), 50 mM KOAc, 10 mM Mg(OAc)_2_, and 1 mM DTT). S1 Figure A shows the results from incubation of SgrAI with ^32^P labeled 22-1 (left) or 22-1-3’ (right), with no activator DNA. Samples were taken from the reaction at different times, before (first lane of the series) and after mixing (1 min, 2 min, 5 min, 10 min, 15 min, 30 min, 45 min, 1 hr, 2 hr, 3 hr). Clearly, the unmodified DNA (22-1, S1 Figure A, left) is cleaved during the course of the incubation, while the modified (22-1-3’S, S1 Figure A, right) is not. The light band (NS, S1 Figure A-B) appearing near the expected cleavage product (C, S1 Figure A-B) runs faster than the expected cleavage product, and is most likely the product of nonspecific cleavage deriving from contaminating nucleases which copurified with the SgrAI enzyme. S1 Figure C shows the result of a similar assay, however with 1 μM PC DNA. Cleavage of unlabeled DNA (22-1, S1 Figure C, right) is accelerated (the incubation times are as in S1 Figure A), while only faint nonspecific cleavage is found in the case of the modified DNA (22-1-3’S, S1 Figure C, left). Figure S1D shows that 22-1, the unmodified DNA, is capable of activating SgrAI (S1 Figure D, left), and also shows that the modified DNA, though capable of activating DNA cleavage of unmodified DNA (S2 Figure), is not capable of activating the cleavage of itself (S1 Figure D, right).
